# Chitosan Composite Membrane with Efficient Hydroxide Ion Transport via Nano‐Confined Hydrogen Bonding Network for Alkaline Zinc‐Based Flow Batteries

**DOI:** 10.1002/advs.202401404

**Published:** 2024-04-15

**Authors:** Jing Hu, Pengfei Wang, Jianbo Hu, Menglian Zheng, Mingdong Dong

**Affiliations:** ^1^ Interdisciplinary Nanoscience Center Aarhus University Aarhus C 8000 Denmark; ^2^ ZJU‐Hangzhou Global Scientific and Technological Innovation Center Hangzhou 311215 China; ^3^ State Key Laboratory of Clean Energy Utilization Zhejiang University Hangzhou 310027 China; ^4^ Institute of Thermal Science and Power Systems School of Energy Engineering Zhejiang University Hangzhou 310027 China; ^5^ Zhejiang Lab Hangzhou 311100 China

**Keywords:** energy storage, hydroxide ion transport, ion‐conducting membrane, zinc‐based flow battery

## Abstract

The development of membranes with rapid and selective ionic transport is imperative for diverse electrochemical energy conversion and storage systems, including fuel cells and flow batteries. However, the practical application of membranes is significantly hindered by their limited conductivity and stability under strong alkaline conditions. Herein, a unique composite membrane decorated with functional Cu^2+^ cross‐linked chitosan (Cts‐Cu‐M) is reported and their high hydroxide ion conductivity and stability in alkaline flow batteries are demonstrated. The underlying hydroxide ions transport of the membrane through Cu^2+^ coordinated nano‐confined channels with abundant hydrogen bonding network via Grotthuss (proton hopping) mechanism is proposed. Consequently, the Cts‐Cu‐M membrane achieves high hydroxide ion conductivity with an area resistance of 0.17 Ω cm^2^ and enables an alkaline zinc‐based flow battery to operate at 320 mA cm^−2^, along with an energy efficiency of ≈80%. Furthermore, the membrane enables the battery for 200 cycles of long‐cycle stability at a current density of 200 mA cm^−2^. This study offers an in‐depth understanding of ion transport for the design and preparation of high‐performance membranes for energy storage devices and beyond.

## Graphical abstract

A chitosan‐based ion‐conducting membrane is meticulously designed and successfully integrated into flow battery applications. The Cu^2+^ cross‐linked hydrogen bonding network of the membrane facilitates OH^−^ conduction by promoting proton transfer (Grotthuss mechanism), resulting in efficient OH^−^ transport. This enables the alkaline zinc–iron flow battery to operate with high efficiency at high current densities, paving the way for efficient ion separation and energy storage.

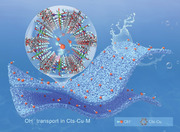

## 1 Introduction

The rapid growth of renewable energy sources, such as wind and solar power, has spurred the demand for advanced energy storage and conversion technologies to mitigate intermittent energy supply and maintain power grid stability.^[^ ^1^ ^]^ Among various promising electrochemical devices for large‐scale renewable energy conversion and storage, flow batteries stand out due to their inherent safety, scalability, and long cycle life.^[^ ^2^ ^]^ Within the flow battery system, ion‐conducting membranes play a critical role, facilitating the rapid transport of charge‐carrying ions while effectively isolating the electrochemical reactions within distinct electrodes.^[^ ^3^ ^]^ The performance of the systems, particularly in terms of efficiency and stability, is profoundly influenced by the properties of membranes.^[^ ^4^ ^]^ Consequently, the development of cost‐effective, high‐performance membranes with exceptional ion conductivity and selectivity is critical for the widespread implementation of electrochemical systems at a large scale. Specifically, in the domain of alkaline flow batteries and fuel cells, there is an emphasis on the demand for superior membrane technology capable of efficiently transporting hydroxide ions and exhibiting exceptional stability.^[^ ^5^ ^]^ Currently, anion exchange membranes have emerged as promising alternatives and gained increasing recognition for their potential in alkaline energy storage devices.^[^ ^6^ ^]^ However, a persistent challenge remains in their conductivity, consistently falling short when compared to their acidic counterparts such as proton exchange membranes.^[^ ^7^ ^]^ This disparity is primarily attributed to the inherently low mobility of hydroxide ions due to the weak basicity of the cation site.^[^ ^8^ ^]^ To address this limitation, extensive research efforts have been directed toward the development of polymers functionalized with diverse cationic groups such as quaternary ammoniums,^[^ ^9^ ^]^ imidazoliums,^[^ ^10^ ^]^ phosphoniums,^[^ ^11^ ^]^ and guanidinium.^[^ ^12^ ^]^ However, despite these advancements, these cationic groups remain susceptible to hydroxide attack, particularly under harsh operating conditions, leading to membrane degradation and compromised long‐term chemical stability.^[^ ^13^ ^]^ As a result, it has remained an ongoing challenge to develop membranes with high hydroxide conductivity and robust chemical stability under the demanding alkaline conditions required for hydroxide ion exchange.

Researchers are increasingly exploring natural polymers as potential solutions to our energy needs, owing to their accessibility and enhanced sustainability compared to synthetic polymer membranes.^[^ ^14^ ^]^ Chitosan, a natural macromolecule, stands out for its good film‐forming properties, affordability, and exceptional mechanical strength, making it a highly attractive material for membrane development.^[^ ^15^ ^]^ The abundance of hydroxyl groups in chitosan confers remarkable hydroxide ion conduction capabilities to chitosan membranes.^[^ ^16^ ^]^ Nevertheless, the substantial swelling and reduced chemical strength of chitosan in aqueous solutions, resulting from its high hydrophilicity, have hindered its practical application as an ion exchange membrane.^[^ ^17^ ^]^ The Cu^2+^ coordination has recently been demonstrated to alter the crystal structure of chitosan, leading to the enhanced mechanical strength of the membrane. The research demonstrated the high hydroxide ion conductivity of chitosan‐Cu and explored the potential of utilizing chitosan in alkaline fuel cells.^[^ ^18^ ^]^ Despite these advancements, further elucidation of the fundamental mechanisms underlying ion transport and selectivity is therefore imperative for the development of ion‐conducting membranes. However, in‐depth studies on the microscopic mechanisms of ion transport in chitosan membranes, particularly the movement of hydroxide ions along ordered hydrogen‐bonded nanostructures, remain scarce, which hindered the broader application of chitosan membranes in the field of ion separation.

**Figure 1:**
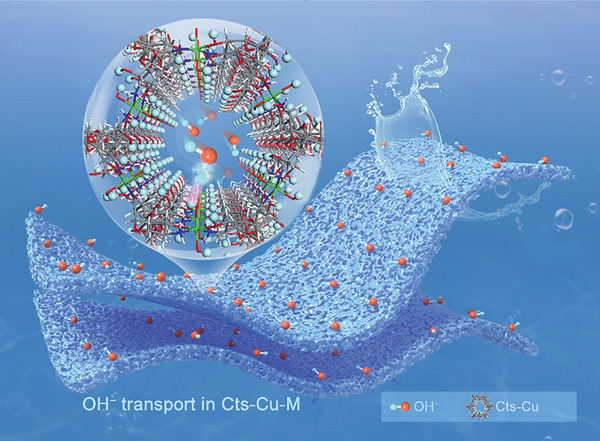
Schematic illustration of hydroxide ions transport in Cu^2+^ cross‐linked chitosan composite membrane.

Herein, we report the development of high‐performance ion‐conducting membranes with enhanced hydroxide ion selectivity and conductivity, specifically tailored for alkaline zinc‐based flow batteries. As schematically shown in **Figure** **1**, by strategically combining Cu^2+^ cross‐linked nano‐confined nanochannels with a robust hydrogen bond network along the membrane surfaces, a high selectivity and superb hydroxide ion conductivity can be achieved. Ab initio molecular dynamics (AIMD) simulations provide unprecedented insights into the transport behavior of OH^−^ ions in the nanoconfinement channels of the composite membrane, revealing the Grotthuss mechanism in membrane nanochannels arise from the synergistic effect between the hydroxyl groups, hydroxide anions, and water molecules within the chitosan chains. As a result, the Cts‐Cu‐M achieves a high hydroxide ion conductivity of 0.068 S cm^−1^ with an area resistance of 0.17 Ω cm^2^. To validate the practicability of the membrane, we constructed and characterized an alkaline zinc–iron flow battery (AZIFB) employing the designed membrane. The AZIFB exhibited exceptional performance, boasting a Coulombic efficiency (CE) of ≈98% and an energy efficiency (EE) of 80% at a current density of 320 mA cm^−2^. Moreover, the membrane demonstrated outstanding stability under harsh alkaline conditions, enabling the AZIFB to maintain ≈200 cycling stability at a current density of 200 mA cm^−2^. These results highlight the selective hydroxide ion transport properties of the chitosan composite membrane and its potential for practical applications in efficient and stable alkaline flow batteries, paving the way for the development of high‐performance membranes.

## 2 Results and Discussion

**Figure 2:**
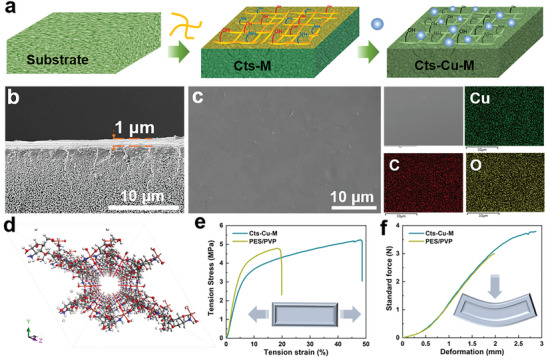
a) Schematic illustration for the preparation of Cts‐Cu‐M. b) The SEM image of the cross‐section morphology of Cts‐Cu‐M. c) SEM image of the surface morphology and corresponding elemental mappings of Cts‐Cu‐M. d) The structural illustration of the Cu^2+^ cross‐linked chitosan chains. e) Puncture resistance mechanical properties of prepared membranes. f) Tension stress–strain curves of prepared membranes.

To obtain a Cts‐Cu‐M composite membrane, as illustrated in **Figure** **2**a of the process scheme, a porous membrane composed of polyethersulfone (PES) polymer molecules and polyvinylpyrrolidone (PVP) polymer prepared through the phase inversion method was employed as the substrate, which is designated as PES/PVP. The morphology of the substrate was tailored through the incorporation of hydrophilic PVP polymer. Detailed analysis conducted via scanning electron microscopy (SEM), as depicted in Figure S1 (Supporting Information), elucidates the smooth surface morphology of the PES/PVP substrate. Its asymmetric morphology and presence of nanoscale pores generally confer high conductivity but low selectivity, rendering it suitable as the support for the composite membrane. Further, chitosan was dissolved in a 4 wt% acetic acid solution and scraped onto the custom‐made PES/PVP substrate using a scrape coating method, then dried naturally overnight to form a continuous chitosan composite membrane (Figure S2, Supporting Information). To improve the mechanical properties of the membrane, the introduction of Cu^2+^ cross‐linking is anticipated to improve the antiswelling properties of the chitosan matrix. Subsequently, the chitosan membrane was immersed in a saturated Na_2_Cu(OH)_4_ solution until it transformed completely into a blue hue, indicating no further color change. This process resulted in the Cu^2+^ cross‐linked chitosan composite membrane, abbreviated as Cts‐Cu‐M.^[^ ^19^ ^]^ Within this membrane, chitosan chains are arranged in a uniform structure, forming a chitosan layer ≈1 µm in thickness, as depicted in Figure 2b,c. The Cts‐Cu‐M exhibited a smooth surface comprising carbon (C), oxygen (O), and copper (Cu) atoms. An asymmetrically cross‐sectional morphology was observed, featuring sponge‐like pores and a skin layer formed during the phase inversion process when the solvent and nonsolvent exchanged (Figure 2b; Figure S3, Supporting Information). Cross‐sectional SEM images of the Cts‐Cu‐M membrane demonstrated a parallelly distributed chitosan layer exhibiting strong adhesion at the interface between the chitosan and the substrate. As illustrated in Figure 2d, the Cu^2+^ ions are coordinated with the amino and hydroxyl groups of the chitosan chains, forming a consistent crystal structure with nanochannels and an abundant hydrogen bond network. This uniform distribution of the Cu^2+^ cross‐linked chitosan layer confers functionality to the membrane.

The composite membrane material should not only endure the piercing force exerted by the electrode mixture/dendrites during battery operation but also satisfy the requirements for physical impact resistance, compression, and tensile strength during the battery assembly process. Figure 2e,f presents the stress–strain curves of membranes for probing their strength and elasticity. The substrate was a polymer with a thickness of about 108 ± 1 µm, which displayed mechanical ability with a tensile strength of 4.8 MPa and the elongation at a break of 19.9%. Chitosan was a semicrystalline polymer when applied in the Cts‐Cu‐M, in comparison, the composite membrane with a thickness of about 110 ± 1 µm exhibited enhanced tensile strength values of 5.2 MPa and elevated elongation at a break of 48.6%. Additionally, the antipuncture performance of the membrane was verified through puncture experiments. For the Cts‐Cu‐M, the maximum load that captured the moment of penetration was 3.79 N, with a corresponding puncture strength of 0.034 N µm^−1^. In comparison, the substrate registered 2.99 N, with a corresponding puncture strength of 0.027 N µm^−1^. The increased tensile strength and puncture strength of composite membranes originate from the superior flexibility of cross‐linked chitosan species. The coordination of Cu^2+^ with the amino and hydroxyl groups of chitosan chains enhances the mechanical strength and stability of the membrane in alkaline solutions. Chitosan's molecular composition boasts a significant presence of amino and hydroxyl groups, imparting commendable flexibility to the material. The relatively spacious gaps between chitosan molecules facilitate the formation of numerous cross‐links, further enhancing its overall flexibility and affording composite membrane‐enhanced mechanical properties.

**Figure 3:**
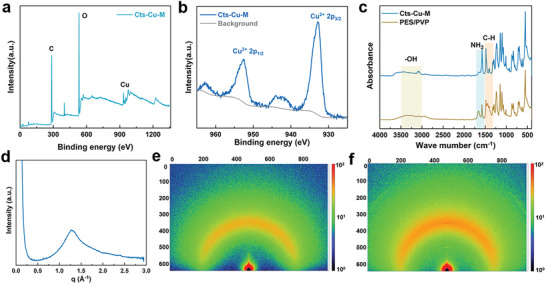
a) The XPS characterization of the membrane surface. b) XPS Cu 2p spectrum of the prepared membranes. c) FTIR spectra of prepared membranes. d) The X‐ray diffraction curves of the Cts‐Cu‐M membrane. e) The corresponding 2D X‐ray diffraction pattern of the Cts‐Cu‐M and f) PES/PVP.

To investigate the structure and chemical bond interactions within the Cts‐Cu‐M membrane, X‐ray photoelectron spectroscopy (XPS) technology was utilized, as illustrated in **Figure** **3**a,b. The Cu 2p spectra of Cts‐Cu‐M revealed two distinctive characteristic peaks at 932.2 and 951.7 eV, affirming the existence of Cu^2+^ within the Cts‐Cu‐M membrane. The existence of Cu–N bonds in Cts‐Cu‐M was validated through N 1s XPS, as illustrated in Figure S4 (Supporting Information). This finding suggests that Cu^2+^ forms cross‐links with the amino groups in the chitosan matrix. Fourier‐transform infrared spectroscopy (FTIR) analysis of Cts‐Cu‐M is presented in Figure 3c. Notably, the broad peak in the range of 3000–3500 cm^−1^ confirms ─OH symmetrical vibrations of hydroxyl groups within the Cts‐Cu‐M structure. A sharp absorption band at 1479 cm^−1^ is attributed to the C–H asymmetric bending vibration of the trimethylammonium group, indicating the presence of quaternary ammonium salt. Additionally, a robust peak related to the primary amine at 1599 shifts to 1650 cm^−1^, corresponding to the secondary amine structure of Cts‐Cu‐M (Figure S5, Supporting Information).^[^ ^20^ ^]^ This shift signifies the successful coordination of amino and hydroxyl groups on the chitosan chains with Cu^2+^, indicating the formation of the Cu–N bond in the Cts‐Cu‐M membrane. These Cu^2+^ ions establish cross‐links between chitosan chains, creating a robust bond network among Cu^2+^ and hydroxyl and amino groups. The structural arrangement is expected to confer the designed membrane with rapid hydroxide ion transport behavior and high alkaline stability.

Two‐dimensional X‐ray diffraction was employed for a detailed investigation into the crystal structure of Cts‐Cu‐M membranes. Grazing Incidence Wide‐Angle X‐ray Scattering (GIWAXS) was utilized to probe the structure features due to the intrinsically low crystallinity of the Cts‐Cu‐M membrane. The X‐ray diffraction profiles of the Cts‐Cu‐M membrane, showing the representative scattering patterns including the typical crescent ring align well with chitosan crystalline interspersed with regions of amorphous structure (Figure 3d).^[^ ^21^ ^]^ Notably, the Cts‐Cu‐M membrane exhibits a low degree of crystallinity, manifesting nonaligned diffraction rings, as illustrated in Figure 3e. In contrast, the crystalline structure of PES/PVP significantly differs from that of the Cts‐Cu‐M membrane. Figure 3f illustrates the unique crystalline pattern of PES/PVP, while different from Cts‐Cu‐M, also exhibits a lower crystalline degree. The formation of hexagonal nanochannels within the Cts‐Cu‐M membrane is attributed to the metal‐ion coordination and its trigonal crystal structure as demonstrated in Figure S6 (Supporting Information).^[^ ^18^ ^]^ After bonding Cu ions with ─NH_2_ and ─OH, the stable structures were obtained by molecular dynamics optimization and DFT optimization, respectively, for subsequent AIMD simulation. This unique arrangement results in the creation of well‐defined ion transport pathways within the membrane. Remarkably, these pathways are lined with free amino and hydroxyl groups, forming an intricate network when cross‐linked by Cu^2+^ ions. This distinctive configuration not only enhances the structural integrity of the membrane but also establishes efficient hydroxide ion transport pathways. The combination of metal‐ion coordination, trigonal crystal structure, and the resulting nanochannels amplifies the potential of the membrane as a robust and efficient component in alkaline flow batteries, opening new avenues for advancements in energy storage technologies.

**Figure 4:**
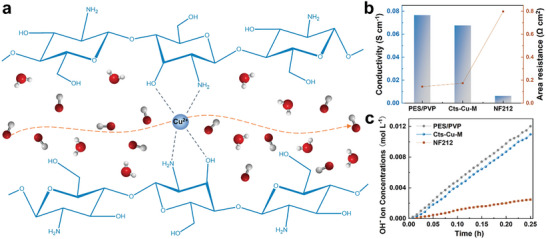
a) Schematic diagram of the OH^−^ transport pathways in Cu^2+^ cross‐linked Cts‐Cu‐M nanochannel. b) The ionic conductivity of different membranes. c) The permeability of hydroxide ions through different membranes.

As the critical parameter of membranes, hydroxide ion conductivity plays an important role in the alkaline flow battery performance. The efficient transport of hydroxide ions through the membrane is essential for maintaining the electrochemical balance and enabling the desired redox reactions.^[^ ^5b^ ^]^ As shown in **Figure** **4**a, the hydroxide ion conducting mechanism in the Cts‐Cu‐M membrane involves a combination of ion solvation and the Grotthuss mechanism in the nano‐confined structure. The unique chemical structure of chitosan, with its hydroxyl groups, provides a favorable environment for the transport of hydroxide ions, making it suitable for applications in alkaline systems.^[^ ^22^ ^]^ The hydroxide ions undergo a Grotthuss‐type transport, where they migrate through a series of hydrogen‐bonded water molecules and chitosan functional groups within the nanochannels of Cts‐Cu‐M, leading to the confined ionic transport. Utilizing the ion transport mechanism outlined above, we evaluate the Cts‐Cu‐M membrane, distinguishing it by its abundant hydroxyl groups and distinct Cu^2+^ cross‐linked structure, rendering it a highly effective OH^−^ conductive membrane. The positive charge imparted by the amino groups of chitosan and the coordinating Cu^2+^ ions is reflected in the zeta potential of 17 mV (Figure S7, Supporting Information), which promotes the selective transport of OH^−^ during the ion exchange process. For better investigating the hydroxide ions OH^−^ conduction capability of the Cts‐Cu‐M membrane, the hydroxide ion conductivity of Cts‐Cu‐M was investigated and compared with the commercial ion‐conducting membrane (Figure 4b; Figure S8, Supporting Information). The conductivity of Cts‐Cu‐M was close to that of the substrate, which was on the order of 10^−2^ S cm^−1^ in 3 mol L^−1^ NaOH solution. The Cts‐Cu‐M demonstrated the hydroxide ion conductivity of 0.068 S cm^−1^ and area resistance of 0.17 Ω cm^2^. This ion conductivity value is much higher than that of commercialized Nafion membrane (NF 212) that is commonly used in flow battery systems. The calculated OH^−^ and Na^+^ transference numbers using the Nernst equation for Cts‐Cu‐M are 0.79 and 0.21, respectively, which suggest that the hydroxide ion serves as the primary charging‐balancing ion for the designed Cts‐Cu‐M. Additionally, the OH^−^ permeation of the membrane was clarified in NaOH solution. As shown in Figure 4c, the OH^−^ permeation rate calculated based on the slope of the line is a bit lower than the PES/PVP substrate and higher than that of NF212, indicating the efficient OH^−^ transport capability of Cts‐Cu‐M. The enhanced ion transfer effect was due to the surface characteristics of Cts‐Cu‐M. The contact angle results are shown in Figure S9 (Supporting Information). It was evident that the Cts‐Cu‐M membrane had a contact angle of 18.4°, indicating the hydrophilicity of the membrane, possibly due to the abundant hydrophilic hydroxyl and amino functional groups on the surface of the Cts‐Cu‐M. The enhanced hydrophilicity made the electrolyte fully wet the membrane surface, which was conducive to the rapid transport of hydroxide ions through the membrane.

**Figure 5:**
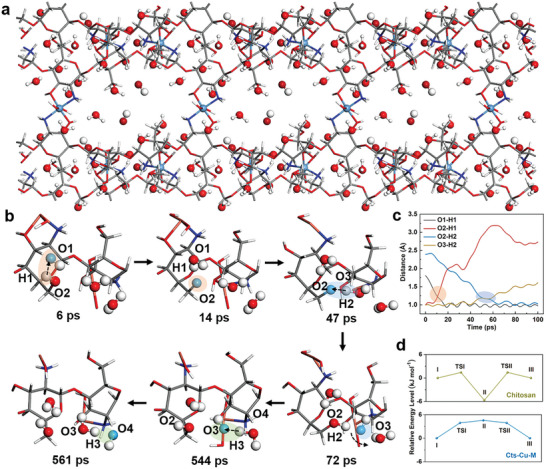
a) The optimized Cu^2+^ cross‐linked chitosan matrix structure for simulating the OH^−^ transport during the AIMD simulation. b) The snapshots responsible for the OH^−^ transport pathways of Cts‐Cu‐M. c) The evolution of O–H distances during the ionic transport process. d) The relative energy level of the ionic transport process. O, H, N, and Cu atoms of the chitosan matrix was shown in red, silver, dark blue, and orange spheres, respectively.

To unravel the transport mechanism of OH^−^ within the Cts‐Cu‐M membrane, ab initio molecular dynamics (AIMD) and density‐functional theory (DFT) simulations were employed, which is a useful tool that enables the investigation of the dynamic behavior of various species under operating conditions.^[^ ^23^ ^]^ The optimized Cu^2+^ cross‐linked chitosan chain model, depicted in **Figures** **5**a and S10 (Supporting Information), revealed a network of six chitosan chains interconnected by Cu^2+^ ions, forming a nano‐confined channel with a high density of hydroxyl groups on its surface. This nanochannel serves as a preferential pathway for the transport of OH^−^ ions. To gain a deeper understanding of the transport behavior of OH^−^, water molecules and hydroxide ions were tracked within the Cu^2+^ cross‐linked chitosan network. Figure 5b and Figure S11 (Supporting Information) showcase chronological snapshots extracted from the AIMD simulation (Movie S1, Supporting Information), revealing that OH^−^ transport can be likened to the reverse movement of protons within nano‐confined chitosan chains. These snapshots aptly demonstrate the efficient migration of OH^−^ ions through the nano‐confined chitosan matrix, a phenomenon attributed to the Grotthuss mechanism, a proton‐hopping process that facilitates OH^−^ transfer.^[^ ^24^ ^]^ In detail, initially, the hydroxide ion resided at the O1H^−^ (pink) position, but as time progressed, the proton H1 (silver) hopped from the O2 atom (hydroxyl group in chitosan chains) to the O1 atom via the Grotthuss mechanism, facilitating the transport of the hydroxide ion from O1H^−^ to O2. This finding demonstrates that the rapid transport of O1H^−^ is facilitated by the hydroxyl groups in chitosan chains (6–14 ps). Subsequently, the energetically unstable O2 rapidly captured an H2 from a nearby water molecule, enabling the transfer of the hydroxide ion from O2 to O3H^−^ (blue position, 47–72 ps). This newly formed O3H^−^ then engaged in translational and rotational motion within the chitosan chains, effectively transporting the hydroxide ion through a vehicular mechanism (72–544 ps). Furthermore, the transport of OH^−^ proceeded sequentially from O3H^−^ to O4H^−^ via the reverse transport of the proton from the neighboring water molecule (H3). These observations clearly indicate that the Grotthuss mechanism contributes significantly to the rapid transport of OH^−^ within the nano‐confined Cts‐Cu‐M. Moreover, the surface ─OH groups (e.g., ─O2H1) of chitosan chains played a critical role in assisting the transport of hydroxide ions, further enhancing the overall efficiency of the transport process. During the transport process of O1H^−^ to O2H^−^, the distance between O1 and H1 diminished, while the distance between O2 and H1 increased, resulting in hydrogen bonding formation. This observation demonstrates that the surface ─OH groups (─O2H1) effectively enhanced the transport of H1. At 47 ps, the observed transfer of H2 from O3 to O2 facilitated the transport of O2H^−^ to O3H^−^, as evidenced by the change in distance (Figure 5c). The enhanced OH^−^ transport barrier through the Cu^2+^ cross‐linked nano‐confined channels was further verified via DFT simulations, as shown in Figure 5d and Figure S12 (Supporting Information). The states I, II, and III represent the OH^−^ moving to the ─OH group, the proton detaching from the ─OH group to the OH^−^, and the O extracting the proton from H_2_O, respectively, corresponding to the states at 6, 14, and 72 ps in Figure 5b. TSI and TSII represent the transfer energy barrier between the nearby two states. Notably, the same model was employed to represent states I and III during the calculation, resulting in equal magnitudes for TSI and TSII. For uncrosslinked chitosan matrix, the energy barrier of proton transfer from H_2_O to O group is 7 kJ mol^−1^.(II‐III) After Cu^2+^ cross‐linking, the energy barrier for proton transfer from the OH group to OH^−^ is 4.5 kJ mol^−1^ (I‐II). This indicates that Cu^2+^ cross‐linking of nano‐confined channels can reduce the OH^−^ transfer energy barrier and promote OH^−^ ion transfer. Overall, the surface ─OH groups of nano‐confined chitosan chains and water molecules play a crucial role in promoting proton dissociation and transfer (Grotthuss mechanism), thereby facilitating efficient OH^−^ transport in Cts‐Cu‐M.

**Figure 6:**
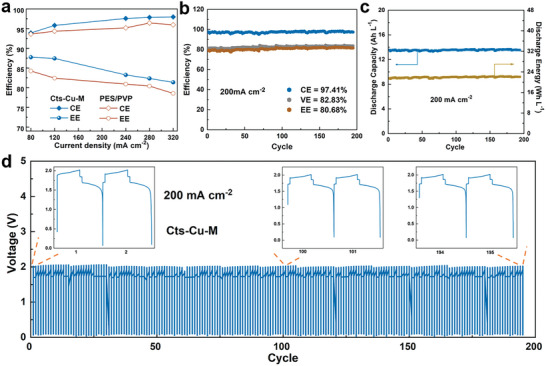
a) Rate performance of AZIFBs employing Cts‐Cu‐M and PES/PVP as the current densities ranging from 80 to 320 mA cm^−2^. b) The cycling performance of Cts‐Cu‐M at the current density of 200 mA cm^−2^. c) Corresponding discharge capacity and discharge energy for each cycle at 200 mA cm^−2^. d) The voltage profiles of AZIFBs employing Cts‐Cu‐M at a current density of 200 mA cm^−2^. Inset: the corresponding detailed voltage profiles.

Due to the efficient hydroxide ion conductivity, the Cts‐Cu‐M is expected to serve as an ideal membrane material for electrochemical energy storage. In the proof‐of‐concept study, we examined the performance of the Cts‐Cu‐M membrane in AZIFBs. **Figure** **6**a illustrates the rate performances of AZIFBs assembled with Cts‐Cu‐M and PES/PVP. As the current density rose from 80 to 320 mA cm^−2^, AZIFBs assembled with Cts‐Cu‐M and PES/PVP both exhibited an increase in CE. The voltage efficiency (VE) and EE slightly decreased due to heightened Ohmic and electrochemical polarizations within the battery. Notably, AZIFBs incorporating Cts‐Cu‐M demonstrated a substantial improvement in both CE and EE compared to those with PES/PVP, which enables an AZIFB to operate at 320 mA cm^−2^, along with an EE of ≈80%. This enhancement is attributed to the Cu^2+^ cross‐linked chitosan layer, which enhances membrane ion selectivity and maintains high hydroxide ion conductivity. In contrast, the AZIFB performance assembled with Nafion 212 exhibited rather low VE and EE at different current densities, primarily attributed to its lower hydroxide ion conductivity (Figure S13, Supporting Information). Figure 6b depicts the cycling stability of AZIFBs with Cts‐Cu‐M at a current density of 200 mA cm^−2^, which exhibited an average CE of 97.41%, EE of 80.68%, and cycling stability over nearly 200 cycles. Furthermore, AZIFBs assembled with Cts‐Cu‐M displayed a stable discharge capacity of ≈14 Ah L^−1^ and a discharge energy of 22 Wh L^−1^ at a current density of 200 mA cm^−2^ (Figure 6c). In contrast, AZIFB assembled with PES/PVP experienced a gradual decline in discharge capacity during cycling (Figure S14a, Supporting Information), attributed to active material crossover, resulting in self‐discharge and decreased capacity (Figure S14b, Supporting Information). Additionally, Cts‐Cu‐M demonstrated a much lower permeation rate for active materials, which endows the AZIFB with a stable voltage profile (Figure 6d). After cycling, the battery was disassembled and the cross‐section and surface morphologies of the CtsCuM were characterized by FE‐SEM. The presence of the residual chitosan layer on the porous substrate served as confirmation of the stability of Cts‐Cu‐M under an alkaline solution (Figures S15–S17, Supporting Information). The synergy of high working current density and elevated open‐circuit voltage bestows the battery with a commendable power density (Figure S18, Supporting Information). In summary, the specially designed composite membrane featuring a Cu^2+^cross‐linked chitosan layer, ensuring an optimal blend of high ion selectivity and conductivity, emerges as a highly promising candidate for alkaline flow batteries.

## 3 Conclusion

In summary, a chitosan‐based ion‐conducting membrane was meticulously designed and successfully implemented in flow battery applications. Comprehensive investigations revealed exceptional hydroxide ion conductivity (6.8 × 10^−2^ S cm^−1^) and alkali stability in robust alkaline solutions. Notably, the theoretical simulations unequivocally demonstrated the Grotthuss mechanism as the predominant transport pathway for hydroxide ions through the Cts‐Cu‐M membrane. The findings unveiled that the nano‐confined Cu^2+^ cross‐linked hydrogen bonding network of chitosan chains facilitates OH^−^ conduction by promoting proton transfer, resulting in remarkable OH^−^ transport efficiency within Cts‐Cu‐M. As a compelling demonstration, an AZIFB assembled with Cts‐Cu‐M exhibited exceptional CE and comparable VE across various current densities, attributed to the membrane's ability to effectively impede active species while facilitating the OH^−^ charge carrier. More significantly, an AZIFB equipped with Cts‐Cu‐M membrane maintained an efficiency exceeding 80% and remarkable cycling stability for ≈200 cycles at a current density of 200 mA cm^−2^. These findings pave the way for the development of versatile membranes with multifunctional ion channels, enabling efficient ion separation and energy storage.

## 4 Experimental Section

### Materials

Sodium ferricyanide and zinc oxide were procured from Shanghai Macklin Biochemical Technology Co., LTD, with a guaranteed analytical grade (AR). Sodium hydroxide and potassium hydroxide, also of analytical grade, were supplied by Sinopharm Chemical Reagent Co., LTD. The carbon felt utilized was GFA3, sourced from CeTech Co., Ltd, with a thickness of 3 mm. Nafion 212 membrane was purchased from E. I. du Pont de Nemours and Company with a thickness of ≈100 µm. Poly(ether sulfone) (PES) was generously provided by Changchun Jilin University Special Plastic Engineering Research. Polyvinylpyrrolidone (PVP) and N, N‐dimethylacetamide (DMAc) were acquired from Aladdin. Chitosan was procured from Shanghai Macklin Biochemical Technology Co., LTD. Copper wires were purchased from Thermo Scientific.

### Preparation of Cts‐Cu‐M

The PES/PVP membrane was meticulously crafted as the substrate using the phase inversion method. PES and a minor quantity of PVP polymers, with a total concentration of 35 wt% (comprising 70 wt% PES and 30 wt% PVP), were initially dissolved in N,N‐dimethylacetamide (DMAc). Subsequently, this solution was evenly cast onto a pristine glass plate and immersed in water, leading to the formation of the porous PES/PVP substrate.

To fabricate the chitosan membrane, 4 g of chitosan was dissolved in 100 mL of a 4 wt% acetic acid aqueous solution at room temperature with continuous stirring overnight. Subsequently, the chitosan solution (0.5 wt%) was carefully cast onto the PES/PVP substrate at room temperature, maintaining a humidity level below 40%. The cast membrane was then air‐dried to yield the final chitosan membrane.

To fabricate the chitosan‐Cu membrane (Cts‐Cu‐M), a blue Cu^2+^‐saturated NaOH solution (Na_2_Cu(OH)_4_) was initially prepared by immersing excess Cu wires in a 20 wt% NaOH solution for a duration of 3 weeks.^[^ ^18^ ^]^ The concentration of saturated Cu^2+^ in the NaOH solution was ≈2 wt%. The chitosan membranes were then immersed in this Na_2_Cu(OH)_4_ solution for 3 days, ensuring saturation, until the blue color of the chitosan material stabilized. Subsequently, the chitosan‐Cu membranes were carefully obtained by rinsing them thoroughly with excess water to eliminate physically adsorbed Cu ions and NaOH. The membranes were then air‐dried to achieve the desired chitosan‐Cu configuration.

### Membrane Characterization

The morphologies and structures of the prepared membranes were examined using field‐emission scanning electron microscopy (SU‐8010, X‐max80, Japan), coupled with energy‐dispersive X‐ray spectroscopy for compositional analysis. Additionally, Fourier infrared spectroscopy (Thermo Scientific Nicolet iS20, USA) and X‐ray photoelectron spectroscopy (Thermo Scientific K‐Alpha, USA) were employed to determine the composition of the Cts‐Cu‐M membranes. Grazing‐Incidence Wide‐Angle X‐ray Scattering (GIWAXS) experiments were conducted using an Xeuss 2.0 instrument with a Cu radiation source to identify the composition and structure of Cts‐Cu‐M. The contact angle of the electrolyte on the membrane surface was measured using a contact angle measuring device (Dataphysics, Germany). Additionally, the zeta potential value of the samples in aqueous solution was determined using a Zeta potential analyzer (Malvern Zetasizer Nano ZS90, UK).

Membrane Conductivity

The membrane conductivity was determined using an Electrochemical Impedance Spectroscopy (EIS) testing station consisting of Solartron SI 1260 and SI 1287 instruments. EIS measurements were conducted over a frequency range of 1 to 100 kHz. For testing, pieces of the membrane were placed between two round titanium plates, each with a diameter of 1.5 cm. The membrane conductivity was calculated using the following equation.

(1) $$\sigma =\frac{L}{R\times A}$$

where σ (S cm^−1^), L (cm), R (Ω), and A (cm^2^) represent the conductivity, the thickness, the resistance, and the effective area of the membrane, respectively.

### Hydroxide Ion Permeation

The OH^−^ ion permeation test was carried out using a diffusion cell. The effective membrane area is 9 cm^2^. For the measurement, 80 mL alkaline solution (3 m NaOH) and 80 mL ultrapure water were added into the draw side and feed side, respectively. The pH of the right side solution was detected at a regular time interval by using Mettler Toledo pH meter mode. All the experiments were conducted at room temperature. The OH^−^ concentration (C_OH_^−^) was calculated from the pH data according to the following equation:

(2) $$pH\;=\;14+lg\left(C_{OH^{-}}\right)$$

### The Ionic Transport Properties

The ionic transport properties of the prepared membranes were investigated using Gamry Interface 3000. The current–voltage (*I*–*V*) profile was recorded when the membrane was sandwiched between two cells soaking with a concentration gradient of 1|3 mol L^−1^ NaOH solution. Two Ag/AgCl references were employed to eliminate the potential drop. Thus, the open‐cell voltage of the device (*V*_0_) is equal to the value of diffusion potential (*V*_d_) resulting from the NaOH concentration gradient, which can be calculated as the following equation.

(3) $$V_{o}=V_{d}=\frac{\mathrm{RT}}{F}\left(t_{Na^{+}}-t_{OH^{-}}\right)\ln\left(\Delta \right)$$

R, T, F, 𝑡_Na_^+^, 𝑡_𝑂𝐻_^−^, 𝑎𝑛𝑑 𝛥 are the gas constant, temperature, faraday constant, Na^+^ transference number, OH^−^ transference number, and activity gradient (the mean ion activity coefficient was considered since the concentration of NaOH solution is high), respectively.

### Mechanical Property

The mechanical stability of the membranes was assessed through a mechanical test (INSTRON 5982), which included the measurement of tensile strength, puncture strength, breaking stress, and elongation at break. Membrane samples, cut to dimensions of 1 × 7 cm, were prepared with five replicates for each sample. The membrane thickness was measured using a spiral micrometer. The samples were securely fixed on a universal testing machine, with clamping widths set at 1 cm on both the left and right sides. The stretch gauge length was 5 cm, and the stretching rate was maintained at 5 mm min^−1^ during the test.

### Electrochemical Measurement and Battery Test

The AZIFB system was assembled by sandwiching the prepared membrane between two carbon‐felt electrodes (GFA3), secured by two graphite plates. The carbon felt electrodes had an area of 25 cm^2^ each. All fittings were held together by eight bolts. The single battery was connected to the electrolyte storage tank and peristaltic pump by a hose. The current collectors were two graphite plates with serpentine flow fields. The negative electrolyte consisted of 60 mL 0.4 mol L^−1^ Zn(OH)_4_^2−^ + 3.8 mol L^−1^ OH^−^, and the positive electrolytes comprised 60 mL 0.8 mol L^−1^ Fe(CN)_6_^4−^ + 3 mol L^−1^ OH^−^. The charge and discharge tests were performed on the CT‐4008 Tn tester (Neware, China).

The electrolyte flow rate was 100 mL min^−1^. This value was calculated according to the following formula:

(4) I=nFQC×min{SoC,1−SoC}

where *I* denotes the current, *n* denotes the number of transfer electrons, *F* denotes Faraday's constant, *Q* denotes the minimum required flow rate, *C* denotes the concentration of reactive ions, and *SoC* denotes the state of charge. To avoid exponentially increased concentration polarization and thus side reaction, we amplified the calculated flow rate data and adopted a flow rate of 100 mL min^−1^. In the charge and discharge tests, we used constant current, the charging process stopped at a fixed time (the corresponding charging time is 9 mins 36 s when the current density was 200 mA cm^−2^), and the discharge voltage ended when it reached 0.1 V.

The polarization curves were measured using the VMP‐3 Electrochemical workstation (Biologic, France). By controlling the charging time, the state of charge (SoC) of the electrolyte was controlled at 20%,50%, and 80% respectively for testing. The test mode was linear sweep voltammetry (LSV). The voltage sweep rate is 10 mV s^−1^, and the initial voltage is the battery's open circuit voltage. The electrolyte flow rate was 100 mL min^−1^.

### Ab Initio Molecular Dynamics (AIMD) and Density‐Functional Theory (DFT) Simulations

The optimization, as well as AIMD simulation, were carried out with the mixed Gaussian plane wave scheme using the CP2K package. The Gaussian and plane waves method as implemented in the QUICKSTEP module was adopted.^[^ ^25^ ^]^ The BLYP functional^[^ ^26^ ^]^ and the DZVP‐MOLOPT‐SR basis set with Goedecker–Teter–Hutter (GTH) pseudo potentials^[^ ^27^ ^]^ were used. During the calculation, the Grimme D3 correction^[^ ^28^ ^]^ with zero damping was applied to account for the dispersion interactions, as well as the plane wave cutoff energy and relative cutoff were 280 Ry and 60 Ry, respectively. The structures were relaxed before performing the AIMD simulation. During the AIMD process, a 16 ps simulation with a time step of 1 fs was performed in the NVT ensemble at 298 K, and controlled by the Nosé–Hoover thermostat.^[^ ^29^ ^]^ The trajectories were recorded at every step to analyze the mean square displacement. The diffusion barrier was calculated by performing the DFT calculation using the Gaussian 16 package.^[^ ^30^ ^]^ The DFT calculations at B3LYP‐D3^[^ ^31^ ^]^/6‐31G**^[^ ^32^ ^]^ level were performed to obtain the optimized minimum‐energy structures, as well as their transition states. In these calculations, the geometries of the clusters and the liquid molecules were allowed to relax. All structures were verified as stationary points on the potential energy surface by performing numerical harmonic vibrational frequency calculations.

## Conflict of Interest

The authors declare no conflict of interest.

## Supporting information

Supporting Information

Supplemental Movie 1

## Data Availability

The data that support the findings of this study are available from the corresponding author upon reasonable request.
