# Supplementary material for: Chitosan Composite Membrane with Efficient Hydroxide Ion Transport via Nano‐Confined Hydrogen Bonding Network for Alkaline Zinc‐Based Flow Batteries
Source: Adv Sci (Weinh). 2024 Apr 15;11(23):2401404. doi: 10.1002/advs.202401404 (PMC11187903; doi:10.1002/advs.202401404)
Supplement: Supplementary file 1 — Supporting Information [file ADVS-11-2401404-s002.pdf]

ADVANCED SCIENCE Open Access

Supporting Information

for *Adv. Sci.*, DOI 10.1002/advs.202401404

Chitosan Composite Membrane with Efficient Hydroxide Ion Transport via Nano-Confined Hydrogen Bonding Network for Alkaline Zinc-Based Flow Batteries

Jing Hu, Pengfei Wang, Jianbo Hu, Menglian Zheng* and Mingdong Dong**

Supporting Information

Chitosan composite membrane with efficient hydroxide ion transport via nano-confined hydrogen bonding network for alkaline zinc-based flow batteries

Jing Hu,* Pengfei Wang, Jianbo Hu, Menglian Zheng,* and Mingdong Dong*

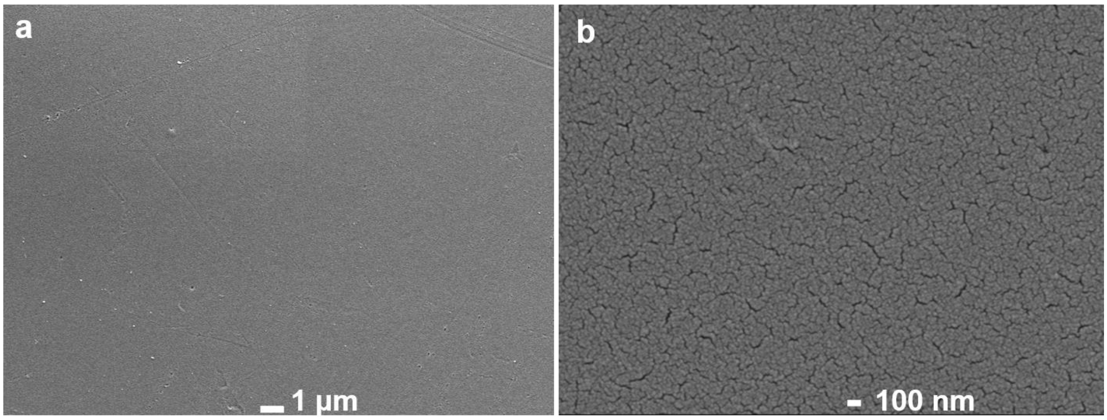

Figure S1. The surface morphology of the PES/PVP substrate.

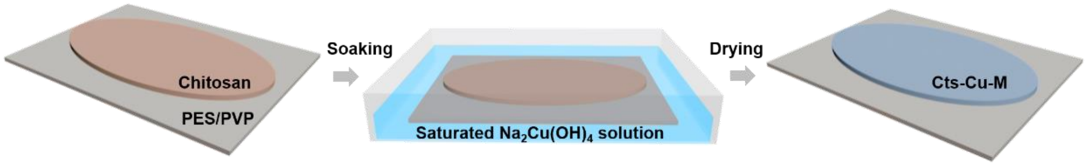

Figure S2. The scheme illustration of preparation process of the designed Cts-Cu-M.

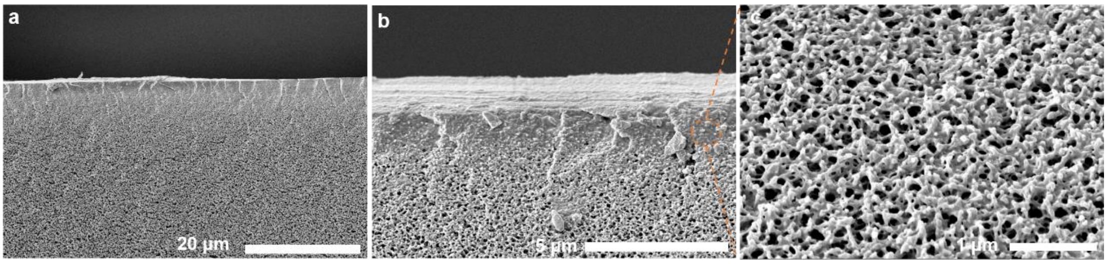

Figure S3. The cross-section morphology of the Cts-Cu-M of different magnifications.

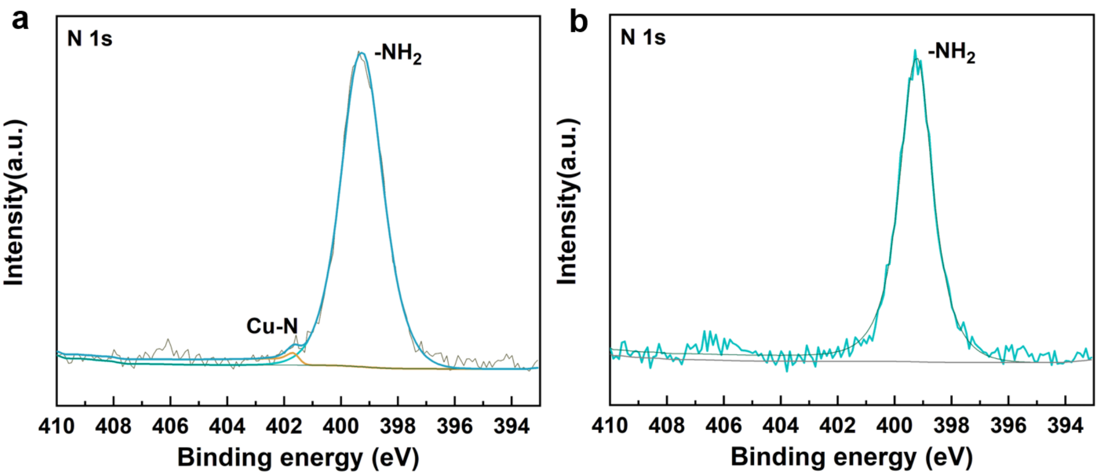

Figure S4. The N1s XPS spectra of the (a) Cts-Cu-M and (b) PES/PVP membrane.

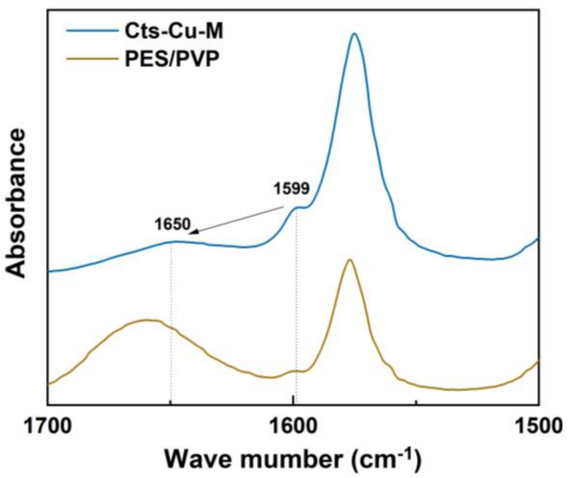

Figure S5. The magnified FTIR spectra of prepared membranes.

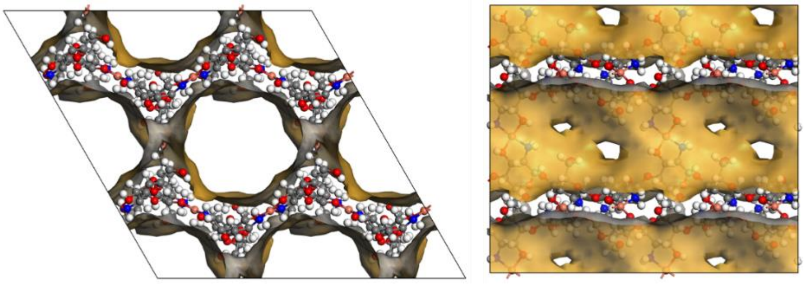

Figure S6. The hexagonal nanochannels of Cts-Cu-M.

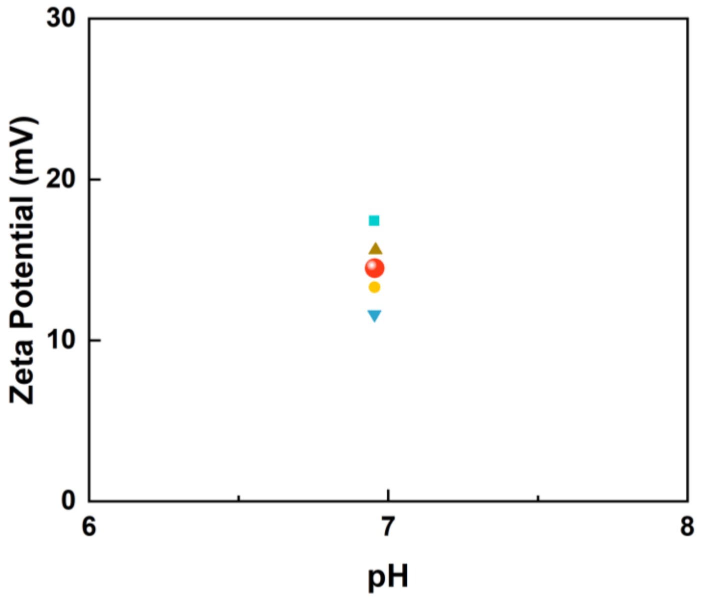

Figure S7. The zeta potential values of Cts-Cu-M at PH=7.

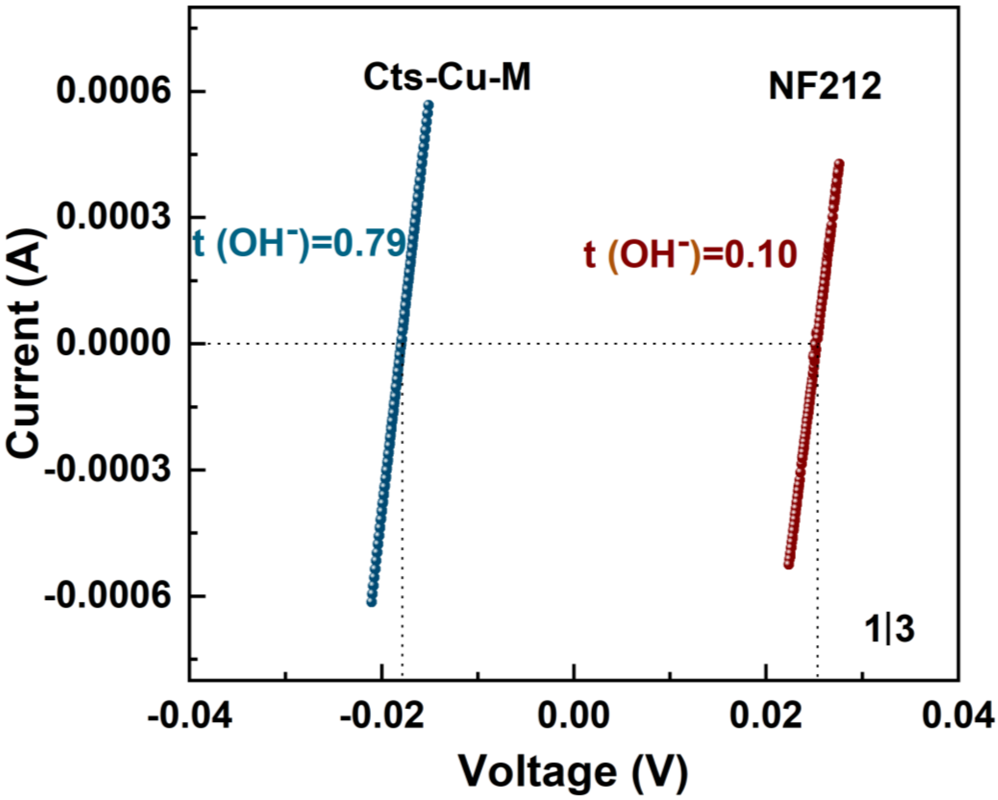

Figure S8. The hydroxide ion transference numbers through different membranes calculated from the current-voltage (I-V) profiles.

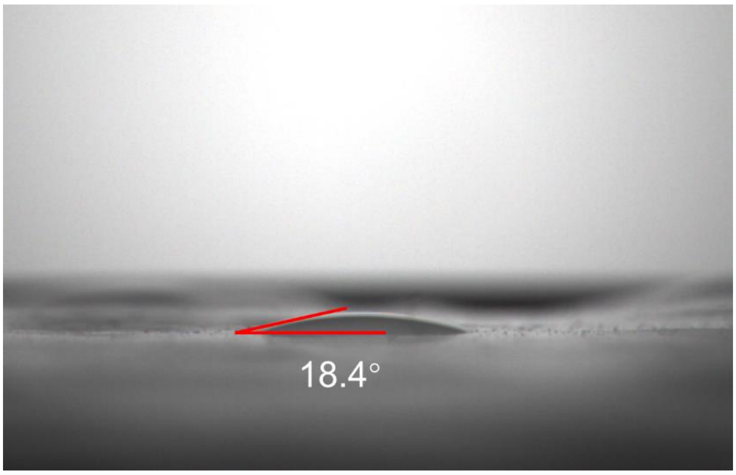

Figure S9. The contact angle result of prepared Cts-Cu-M under 3M NaOH solution.

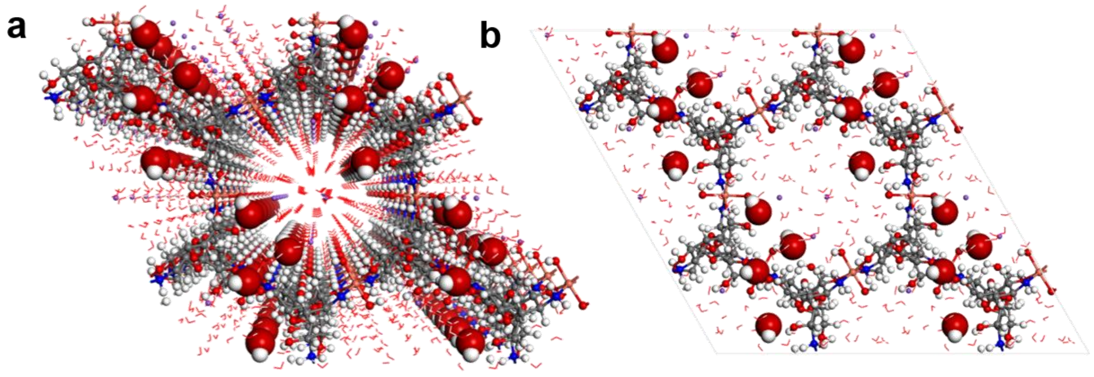

Figure S10. The corresponding models of different perspectives of nano-confined channels for simulating the OH^{-} transport during the AIMD simulation.

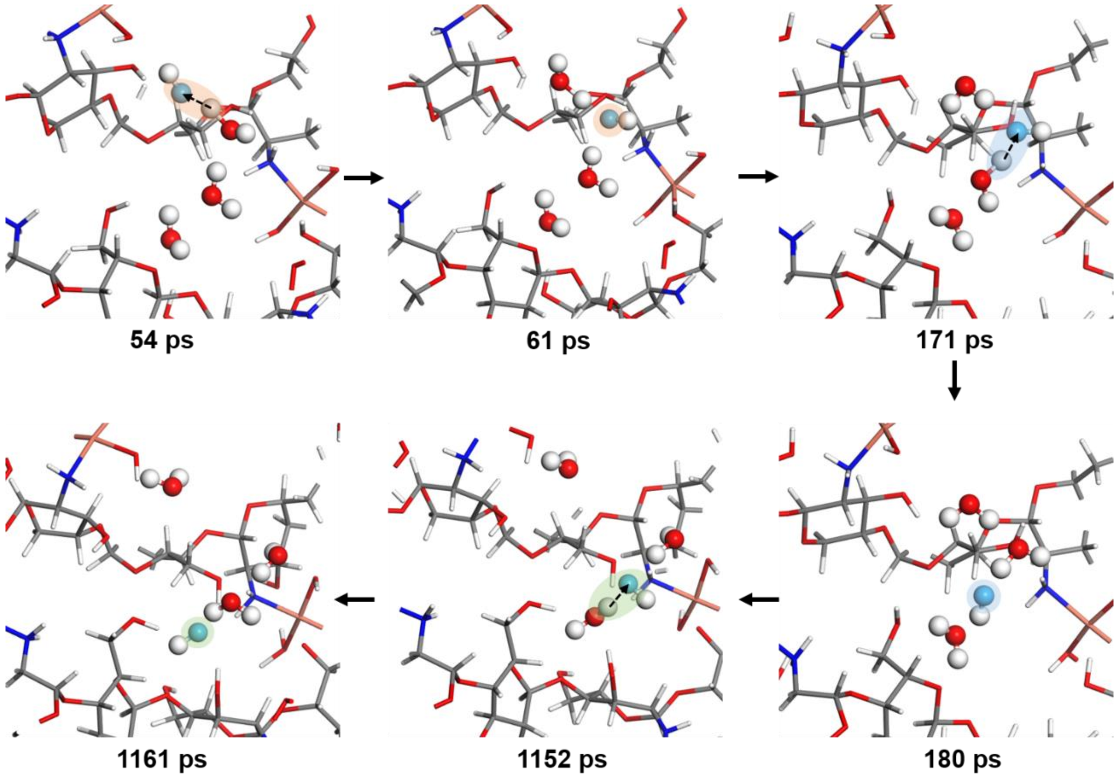

Figure S11. The snapshots extracted from the AIMD simulation, revealing the OH^- transport within Cts-Cu-M.

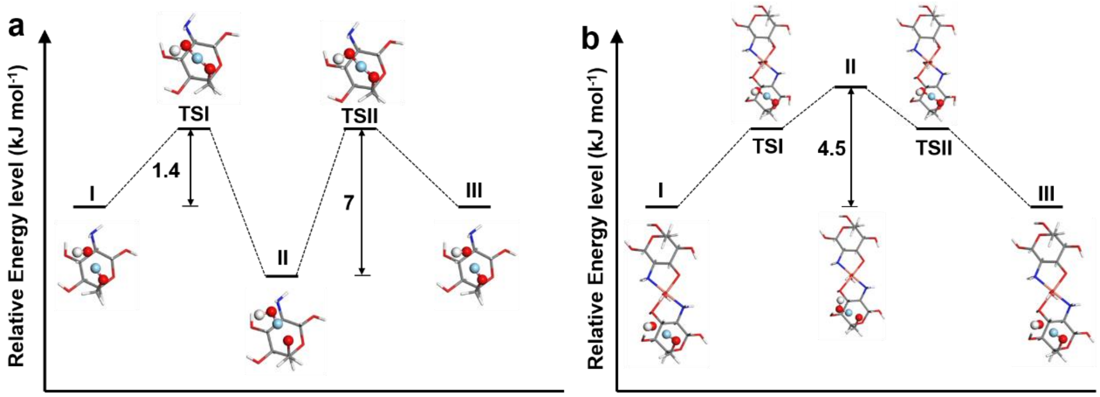

Figure S12. The energy barrier of proton transfer for (a) uncrosslinked chitosan matrix and (b) Cu^{2+} cross-linked nano-confined channels of Cts-Cu-M.

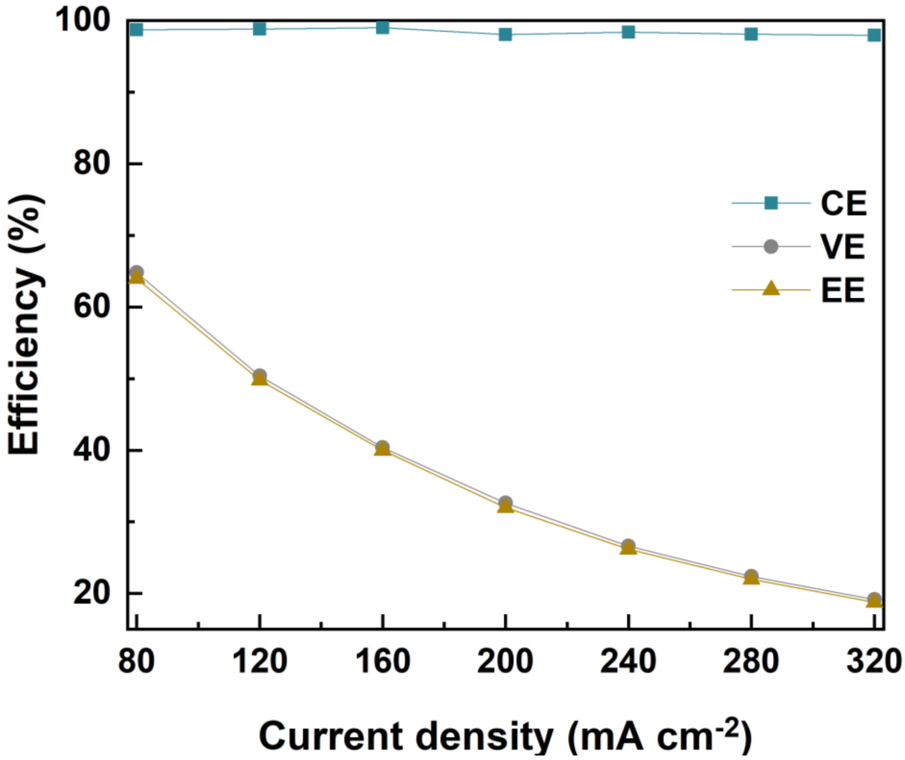

Figure S13. The AZIFB performance assembled with Nafion 212 at different current densities.

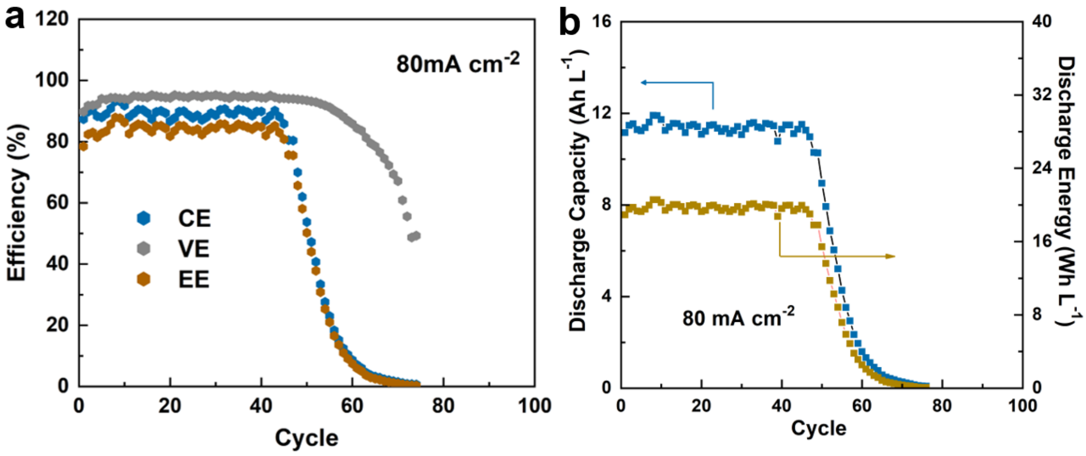

Figure S14. (a) The cycling performance of AZIFB assembled with PES/PVP at the current density of 80 mA cm^{-2}, and (b) corresponding discharge capacity and discharge energy for each cycle at 80 mA cm^{-2}.

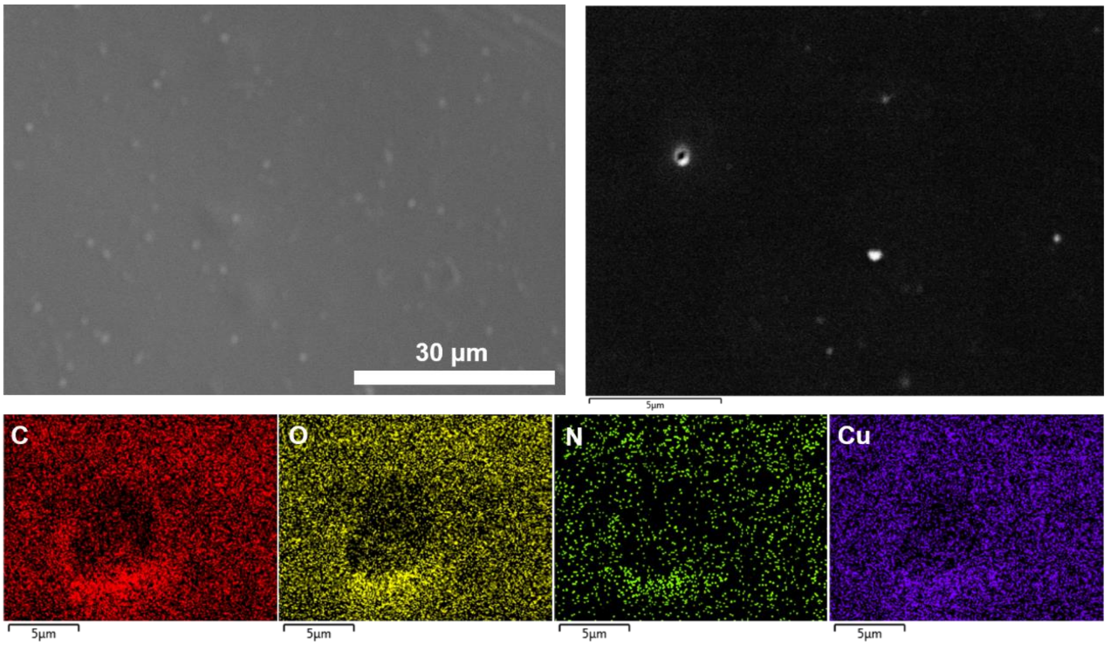

Figure S15. The SEM morphology of Cts-Cu-M after 200 cycles of cycling at the current density of 200 mA cm^{-2}.

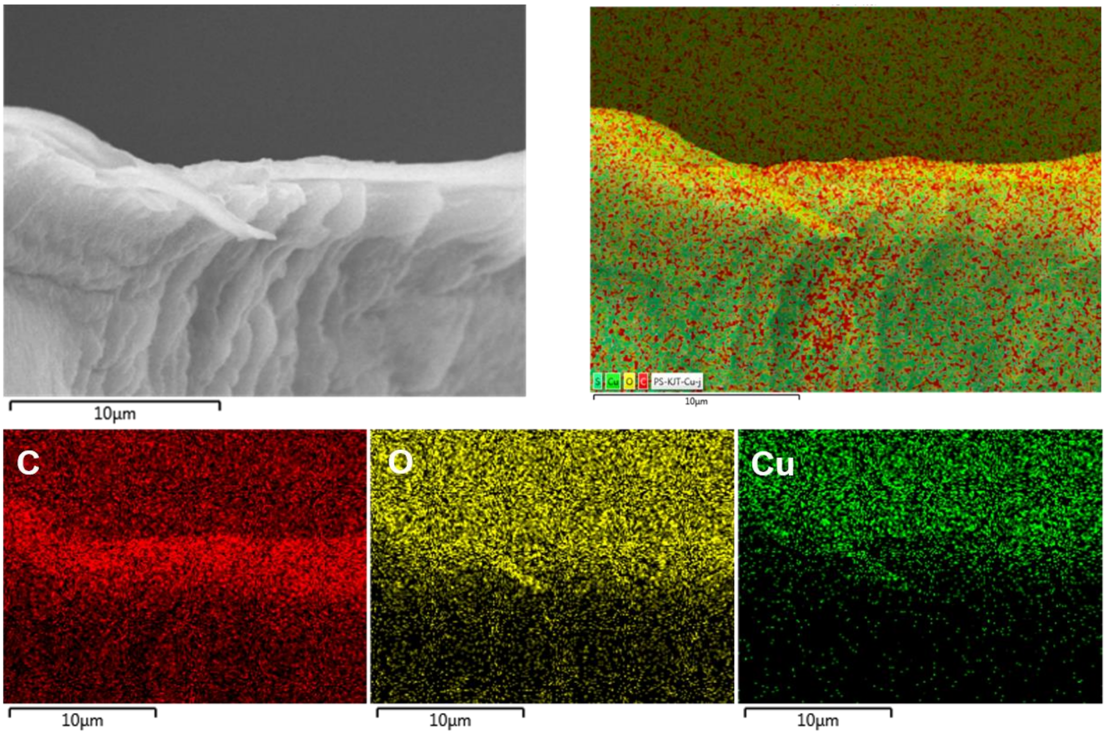

Figure S16. The cross-section morphology of Cts-Cu-M after 200 cycles of cycling at the current density of 200 mA cm^{-2}.

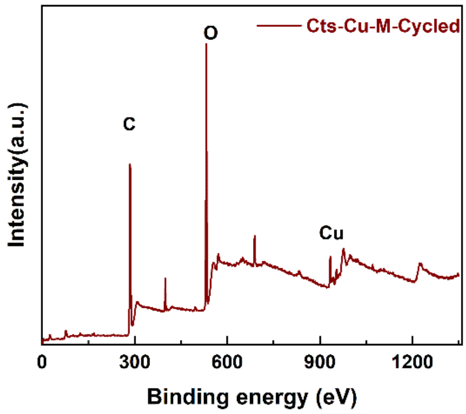

Figure S 17. The XPS characterization of Cts-Cu-M-Cycled membrane surface.

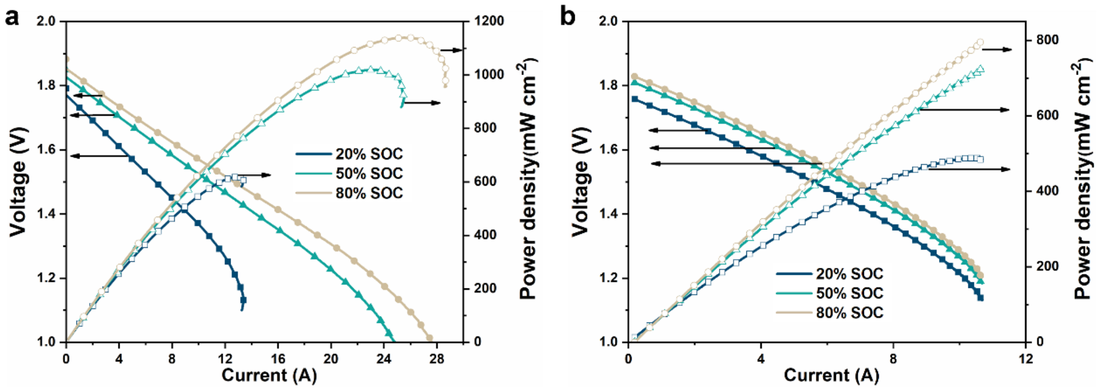

Figure S18. The polarization curves of AZIFB at 80% SOC, 50% SOC, and 20% SOC employing (a) Cts-Cu-M membrane, and (b) PES/PVP.
